# Doctor

**Published:** 1886-11

**Authors:** 


### "DOCTOR."

Perhaps there is nothing in American professional ethics which strikes the educated foreigner with such utter ludicrousness as the indiscriminate use of the title "doctor." The term is derived from the Latin *Docere*, to teach, and in its proper sense is applied to one who is a teacher, an instructor, a learned man. When it is conferred upon a graduate in law or letters, the recipient is thereby licensed as a teacher. In medicine its use is more general, and it is conferred in course as a legal qualification for the practice of physic. But no one is rightly entitled to its use unless he shall have had the doctorate conferred upon him by a legally constituted school. In most of the countries of Europe, a man who styles himself "Doctor" without having gone through a regularly prescribed course of study and received the proper diploma, is liable to fine and imprisonment for swindling the public. It is an honorable distinction, and the use of the title is jealously guarded.

In this country, however, it is not a title of honer, but is commonly used simply to distinguish a man's avocation. All who follow the healing art, whether regularly or irregularly, honorably or dishonorably, claim to be "doctors." There are horse "doctors," cattle "doctors," and dog "doctors," and in the west we frequently hear of a man who has taken up "doctoring" as a means of livelihood. It need not be said that this is a misuse of the term, and that its practice has, in medicine at least, debased the meaning. The term has, by its vulgar use, become akin to that of "Professor," which is claimed by a majority of the tenth-rate music teachers of the country, by negro-minstrel banjoists, and by colored whitewashers, until the most of those to whom rightfully belongs the honorable title disdain to use it.

But the "doctor" is nowhere so flagrantly abused as among dentists. Every fourth-rate, cross-roads, peripatetic tooth-puller, puts "doctor" on his sign and cards, and is exceedingly solicitous of being addressed by that title. It is no longer a name of distinction, and so common is it in application that if a foreigner comes among us who does not hold the doctorate, and who is himself desirous of following the custom of his own country, he is constantly embarrassed by being, on every side, dubbed "doctor," *ad nauseam*.

This is a great country for tinsel titles, but of all the cheap designations that of "doctor" is fast becoming the most worthless.

